# A minimal bile salt excretory pump promoter allows bile acid-driven physiological regulation of transgene expression from a gene therapy vector

**DOI:** 10.1186/s13578-022-00803-9

**Published:** 2022-05-31

**Authors:** Javier Martínez-García, Manuela Molina, Leticia Odriozola, Angie Molina, Gloria González-Aseguinolaza, Nicholas D. Weber, Cristian Smerdou

**Affiliations:** 1 Division of Gene Therapy and Regulation of Gene Expression, Cima Universidad de Navarra, Av. Pio XII 55, 31008 Pamplona, Spain; 2 Instituto de Investigación Sanitaria de Navarra (IdISNA), Pamplona, Spain; 3 Vivet Therapeutics S.L., Calle Arcadio María Larraona, 1 – 2ª planta, 31008 Pamplona, Spain

**Keywords:** BSEP promoter, Bile acids, AAV, Cholestatic diseases, Gene therapy

## Abstract

**Background:**

Bile acid (BA) homeostasis is mainly regulated by bile salt excretory pump (BSEP), a hepatocyte transporter that transfers BAs to the bile. BSEP expression is regulated by BA levels through activation of farnesoid X receptor transcription factor, which binds to the inverted repeat (IR-1) element in the BSEP promoter. Gene therapy of cholestatic diseases could benefit from using vectors carrying endogenous promoters physiologically regulated by BAs, however their large size limits this approach, especially when using adeno-associated viral vector (AAV) vectors.

**Results:**

We evaluated the functionality and BA-mediated regulation of minimal versions of human and mouse BSEP promoters containing IR-1 using AAV vectors expressing luciferase. Unexpectedly, a minimal mouse BSEP promoter (imPr) showed higher BA-mediated expression and inducibility than a minimal human promoter (ihPr) or than full-length BSEP promoters in human hepatic cells. In addition, in mice receiving an AAV8 vector carrying imPr promoter-driven luciferase expression was efficiently regulated by administration of a BA-enriched diet. Interestingly, this vector also expressed significantly higher luciferase levels in *Abcb4*^*−/−*^ mice, which have high levels of BAs, compared to wild type mice, or to mice receiving a vector containing the luciferase gene downstream of the constitutive alpha-1 antitrypsin promoter. In contrast, the AAV vector containing ihPr showed very low luciferase expression with no inducibility. Finally, we optimized imPr by adding three IR-1 repeats at its 5′ end. This new promoter provided higher levels of luciferase than imPr both in vitro and in vivo.

**Conclusions:**

The imPr could represent a useful tool for gene therapy approaches in which physiological BA regulation is desired.

**Supplementary Information:**

The online version contains supplementary material available at 10.1186/s13578-022-00803-9.

## Background

Bile acids (BAs) are synthetized by the liver, and secreted into the bile through the bile salt export pump (BSEP) present on the biliary canaliculi of hepatocytes [1]. Phospholipid transporters multidrug resistance protein 3 (MDR3) and familial intrahepatic cholestasis 1 (FIC1) are also involved in the process of bile synthesis, and their functionality creates an asymmetrical distribution of phospholipids in the hepatocyte membrane bilayer [2]. FIC1 produces an enrichment of phosphatidylserine and phosphatidylethanolamine on the inner leaflet of the plasma membrane, while MDR3 is a floppase that transports phosphatidylcholine into the bile where it forms mixed micelles with BAs and cholesterol. These mechanisms exert a protective role against bile toxicity in canaliculi and biliary epithelium. Most BAs are reabsorbed in the ileum and circulate back to the liver through the bloodstream. BAs are important for the elimination of cholesterol and solubilization of ingested fat and fat-soluble vitamins, facilitating their digestion and absorption. However, accumulation of BAs in the liver can be very toxic, something that happens in diseases resulting from impaired BSEP function or expression, like progressive familial intrahepatic cholestasis type 2 (PFIC2), intrahepatic cholestasis of pregnancy, drug-induced cholestasis, and liver cancer [3]. In other diseases in which the transport of phospholipids is impaired, like PFIC3 or PFIC1, due to mutations in MDR3 and FIC1 coding genes, respectively, the homeostasis of BAs is also altered, leading to hepatic toxicity. The life expectancy and quality of life of most patients with PFIC are lower mainly due to the lack of effective treatments.

Both BSEP and MDR3 are membrane proteins with a complex physiological regulation. BSEP functionality, for example, is important to prevent accumulation of BAs in hepatocytes. The master regulator of BA homeostasis in liver is the farnesoid X receptor (FXR), a member of the nuclear receptor superfamily of ligand-activated transcription factors. After being activated by BAs, FXR can bind to response elements (FXRE) present in promoters of different genes, and activate or inhibit them. Genes activated by FXR include canalicular transporters like BSEP, MDR3, and multidrug resistance-associated protein 2, all of which are involved in bile production [4–6]. FXR can also inhibit BA synthesis in hepatocytes by inducing the transcription of the small heterodimer partner (SHP), which represses expression of cholesterol 7ɑ-hydroxylase (Cyp7a1), an enzyme involved in the synthesis of BAs from cholesterol [7–9].

Although two FXR genes have been identified (FXRα and FXRβ), only FXRα is expressed in humans. FXRα has four different isoforms (FXRα1-4) that are derived from alternative splicing. FXRα1 and FXRα2 are predominantly expressed in the liver [10]. The BSEP promoter (BSEP-Pr) contains an FXRE formed by two inverted repeats separated by one nucleotide (IR-1) to which FXRα can bind as a heterodimer with 9-cis-retinoic acid receptor (RXR) [11]. Other elements in BSEP-Pr that are important for regulation of this gene include the half ER2 (everted repeat separated by two nucleotides) motif, required for maximal FXR transactivation, and the receptor homolog 1-responsive element (LRHRE) which is regulated by the liver receptor homolog 1 (LRH-1) transcription factor [12].

Canonical gene therapy approaches generally utilize promoters that differ from endogenous gene regulation sequences for multiple reasons, such as increasing expression levels, inducing tissue- or cell-specific transactivation, and sequence size limits. The possibility of regulating gene expression according to BA levels could be very useful for some gene therapy applications, especially for those targeting diseases in which the aim is the restoration of BA homestasis, such as PFIC2 and PFIC3. This type of therapy could benefit from the use of BA-controlled promoters, such as BSEP-Pr. However, this promoter is very large (> 2.4 kb) [13] significantly limiting its use in the context of some gene therapy vectors such as those derived from adeno-associated virus (AAV) which have a small packaging capacity [14]. Thus, a smaller promoter with the same functionality would represent an attractive approach for the development of AAV-based gene therapy vectors expressing genes involved in BA homeostasis, many of which have a very large size [15].

To evaluate the possibility of using gene therapy vectors with BA-controlled regulation, we have developed AAV vectors expressing the luciferase reporter gene under the control of minimal versions of human and mouse BSEP-Pr. These vectors showed a potent inducibility in human and murine hepatic cells when supplemented with BAs. Gene expression induction was also observed in vivo when these vectors were administered to either mice fed with a BA-enriched diet or to *Abcb4*^*−/−*^ mice, which naturally have high BA levels in serum [16].

## Results

### Analysis of luciferase expression from minimal bile salt inducible promoters in human and mouse cell lines

**Fig. 1:**
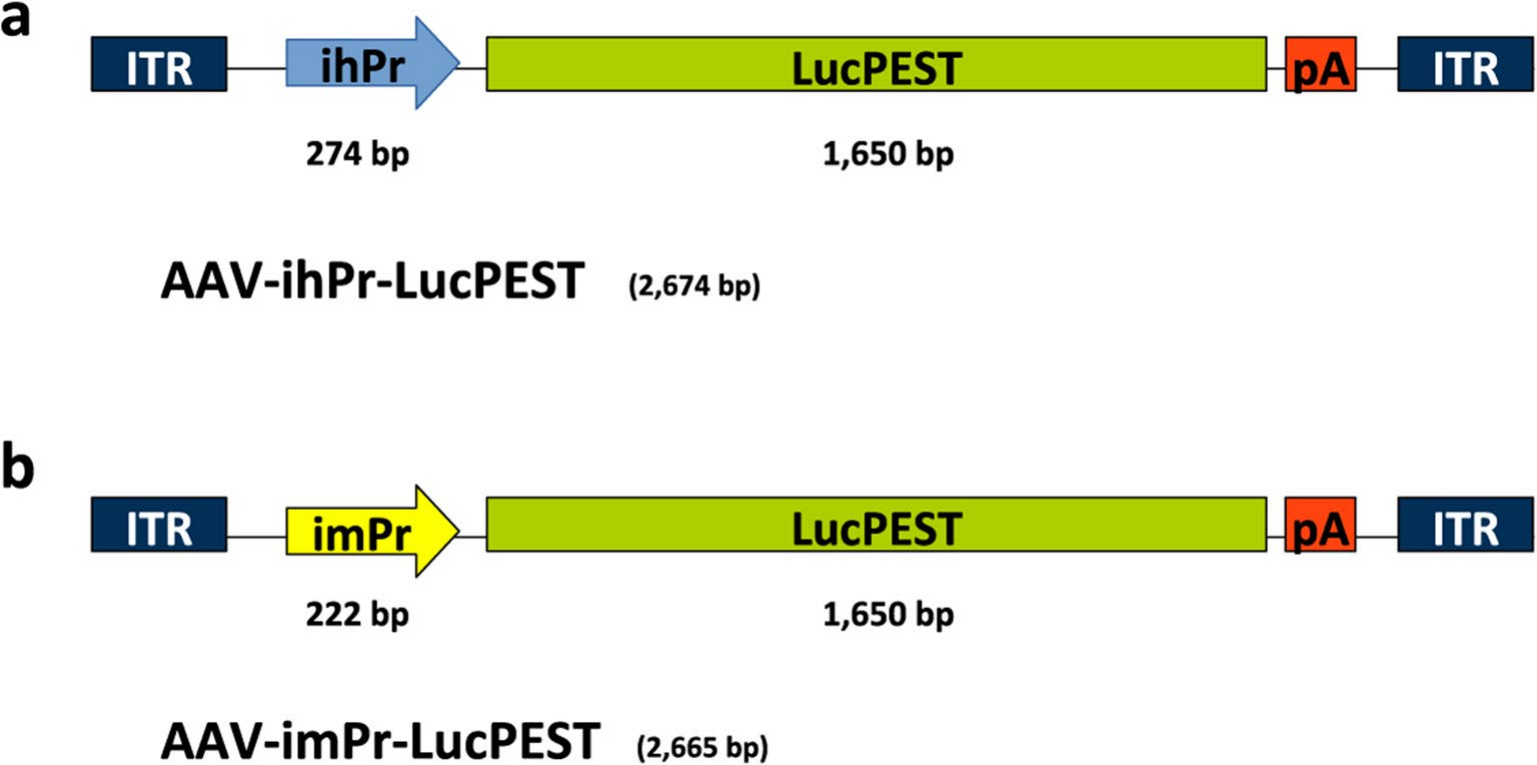
AAV vectors expressing LucPEST downstream of BSEP minimal promoters. Schematic representation of AAV vectors containing a BSEP minimal promoter from human (ihPr) (**a**) and mouse (imPr) (**b**) origin. ITR: inverted terminal repeats; LucPEST: destabilized firefly luciferase sequence; pA: synthetic polyadenylation signal

We evaluated the use of BSEP minimal promoters from both human and mouse origin using a destabilized firefly luciferase gene (LucPEST) as a reporter gene. For the human version (ihPr), we used the last 145 nucleotides (nt) of the human BSEP promoter followed by the first 86 nt of the human BSEP mRNA 5´ untranslated region (UTR) (Additional file 1: Fig. S1a). This region was chosen because it contains the IR-1 element (5´-GGGACATTGATCCT-3´) at positions -63/-50 from the transcription initiation site and has previously shown to be efficiently transactivated by FXR [4]. The murine version (imPr), derived from mouse sequences, was equivalent except it had only the first 77 nt of the mouse BSEP mRNA 5´ UTR (Additional file 1: Fig. S1b). We subcloned these minimal promoters upstream of the LucPEST gene in AAV vector plasmids, generating pAAV-ihPr-LucPEST and pAAV-imPr-LucPEST (Fig. 1). These plasmids have an approximate size of 2.6 kb, which is within the packaging limit of AAV vectors [14].

**Fig. 2:**
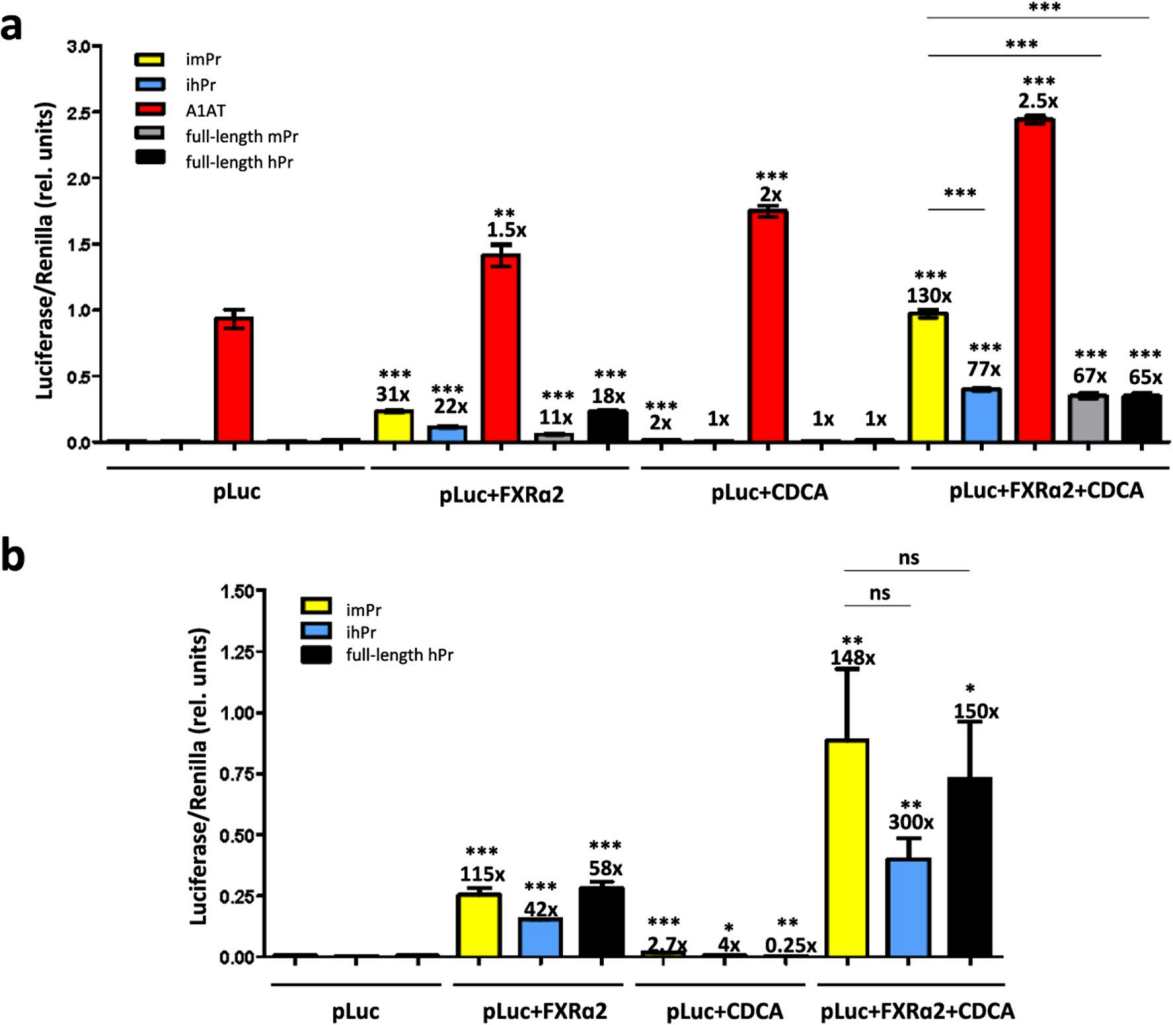
Bile acid induction of luciferase expression in human hepatic cell lines. HepG2 (**a**) and Huh-7 (**b**) cells were transfected with plasmids expressing LucPEST downstream of the indicated promoters with or without co-transfection of the human FXRα2 isoform and with or without incubation with chenodeoxycholic acid (CDCA). All samples were tested in triplicates and data are presented as mean ± SEM of relative (rel.) units (Luciferase units sec^−1^/Renilla units sec^−1^). The fold induction of each condition relative to cells transfected only with the various luciferase plasmids (pLuc) is indicated above each bar, as well as the statistical analysis comparing these two conditions. Other comparisons are shown by horizontal bars. *p < 0.05; **p < 0.01; ***p < 0.001; ns: not significant. imPr, pAAV-imPr-LucPEST; ihPr, pAAV-ihPr-LucPEST; full-length mPr, pAAV-mBSEPpr-LucPEST; full-length hPr, p-2563/ + 4-Luc; A1AT, pAAV-A1AT-LucPEST

**Fig. 3:**
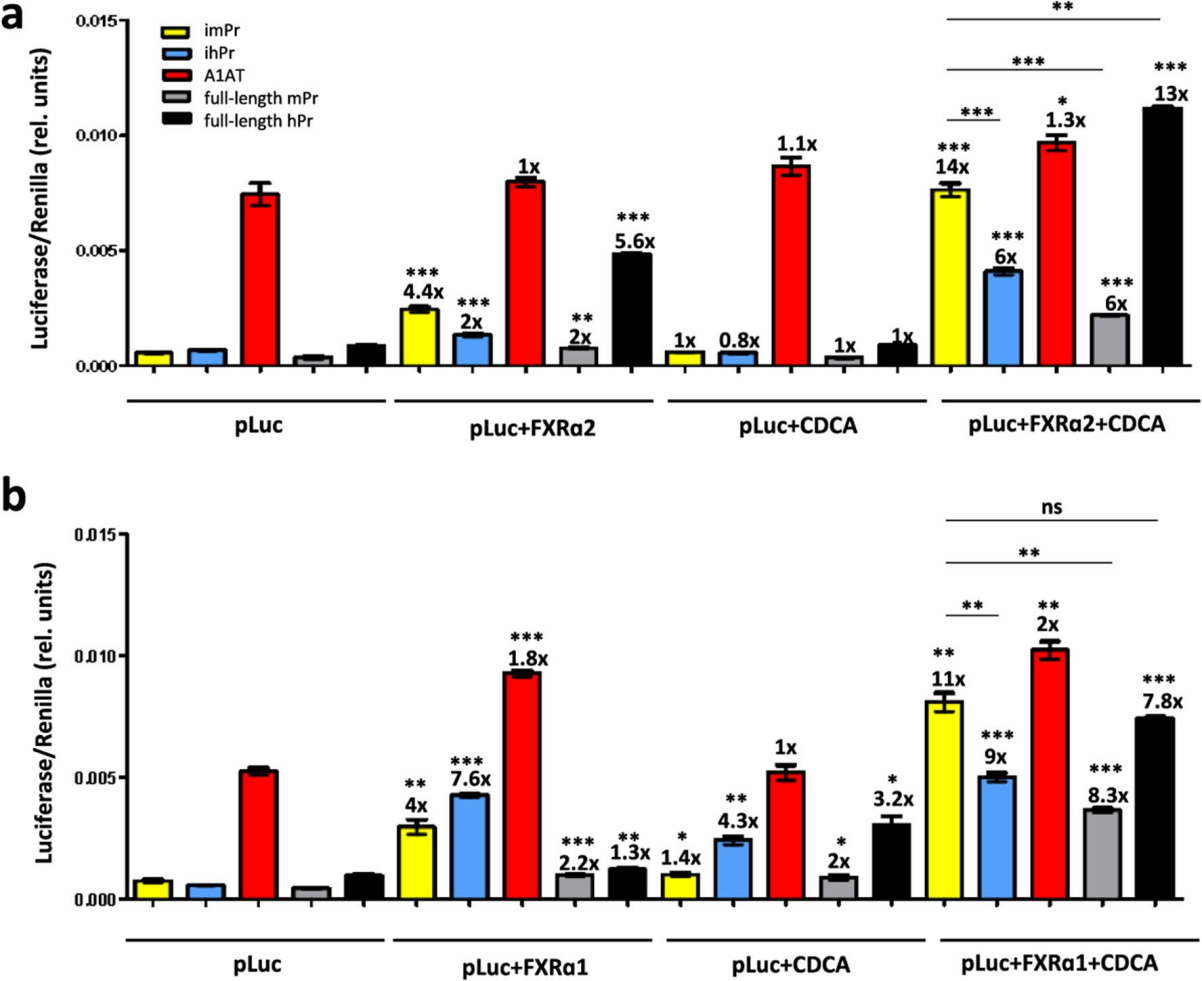
Bile acid induction of luciferase expression in a murine hepatic cell line. Hepa 1–6 cells were transfected with plasmids expressing LucPEST downstream of the indicated promoters with or without co-transfection of human FXR isoforms (FXRα2 in **a** and FXRα1 in **b**) and with or without incubation with CDCA. All samples were tested in triplicates and data are presented as mean ± SEM of relative (rel.) units (Luciferase units sec^−1^/Renilla units sec^−1^). The fold induction of each condition relative to cells transfected only with the various luciferase plasmids (pLuc) is indicated on top of each column, as well as the statistical analysis comparing these two conditions. Other comparisons are shown by horizontal bars. *p < 0.05; **p < 0.01; ***p < 0.001; ns: not significant

To perform an in vitro comparative analysis of promoter inducibility, we transfected human and mouse hepatic cell lines with pAAV-imPr-LucPEST or pAAV-ihPr-LucPEST plasmids, or with control plasmids expressing LucPEST from full-length mouse and human BSEP promoters (pAAV-mBSEPpr-LucPEST, p-2563/ + 4-Luc, respectively), and from the constitutive alpha-1 antitrypsin (A1AT) promoter (pAAV-A1AT-LucPEST), together with plasmids expressing either human FXRɑ1 or FXRɑ2. We included FXR-expressing plasmids in *trans* to provide sufficient levels of FXR for optimal inducibility [4, 10]. Twenty-four hours after transfection, cells were supplemented with chenodeoxycholic acid (CDCA), a bile acid that has been previously shown to activate BSEP expression [4, 17, 18]. We observed the highest CDCA-mediated luciferase induction in cells co-transfected with BSEP promoters and FXR expressing plasmids (Figs. 2, 3 and Additional file 1: Figs. S3, S4). The two FXR isoforms were equally efficient in mouse cells (Fig. 3), while FXRɑ2 was more efficient in human cells (Fig. 2 and Additional file 1: Figs. S3, S4), as has been previously reported [10]. Interestingly, in both human and mouse cell lines, imPr was able to provide higher inducibility and absolute expression levels compared to ihPr, regardless of the FXR isoform that was used (Figs. 2, 3). In addition, imPr also provided higher expression when compared to human and murine endogenous full-length promoters. This was an unexpected result, especially in human cells transactivated with human FXR isoforms, since in these cells human full-length BSEP promoter showed a similar inducibility as mouse full-length BSEP promoter (Fig. 2 and Additional file 1: Fig. S4).

### A bile acid enriched diet can induce luciferase expression in C57BL/6 mice transduced with AAV-imPr-LucPEST

**Fig. 4:**
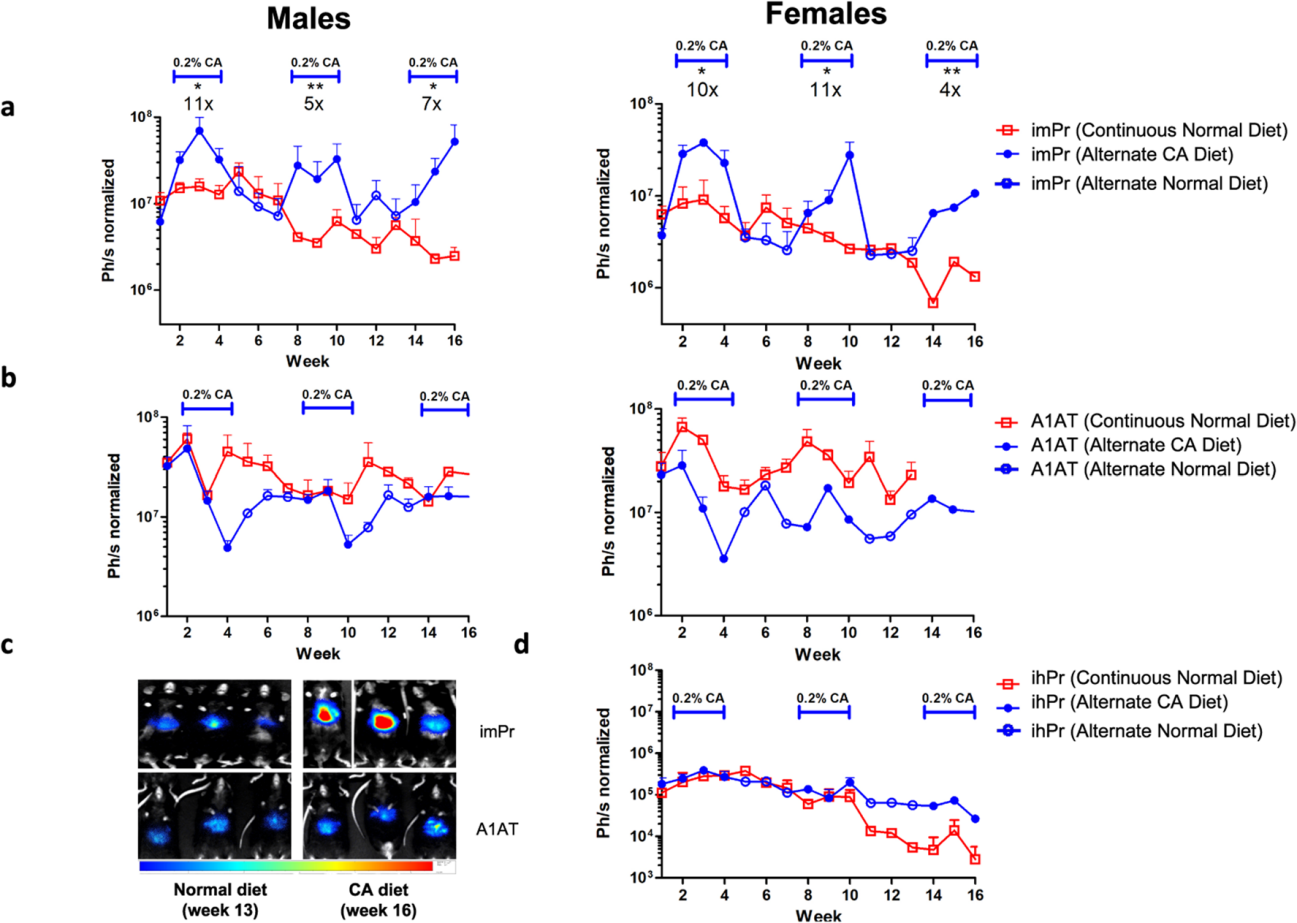
Bile acid induction of minimal BSEP promoters in wild-type (WT) mice. C57BL/6 male and female mice were administered 3 × 10^12^ VG/kg of AAV8-imPr-LucPEST (**a**), AAV8-A1AT-LucPEST (**b**), or AAV8-ihPr-LucPEST (**d**) and received either normal diet (red open squares) or a diet supplemented with 0.2% cholic acid (CA) (blue closed circles) alternating with normal diet (blue open circles) as indicated in the figure. Luciferase expression was measured in live mice at the indicated times (n = 3 for each gender except in mice receiving AAV-ihPr-LucPEST where n = 2, only tested in females). **c** Representative bioluminiscence images of C57BL/6 male mice administered with AAV8-imPr-LucPEST and AAV8-A1AT-LucPEST (n = 3) that received alternating CA/normal diets. Images were taken at week 13 before the last CA cycle and at week 16 after 3 weeks of CA diet in the third cycle. Data are shown as mean + SEM of photon (Ph) units per second normalized with background signal. Statistical comparisons were calculated comparing the average luciferase expression during each CA cycle and its precedent normal diet cycle using a paired T test. *p < 0.05; **p < 0.01. Fold induction in each CA cycle was calculated by dividing the maximum expression under CA induction by basal expression prior to CA administration. imPr, AAV8-imPr-LucPEST; ihPr, AAV8-ihPr-LucPEST; A1AT, AAV-A1AT-LucPEST

In order to analyze whether imPr and ihPr could also be induced in vivo with bile acids, we generated AAV8 viral particles carrying imPr-LucPEST and ihPr-LucPEST expression cassettes, as well as A1AT-LucPEST as a non-inducible control, as described in Methods. Four-week-old C57BL/6 mice were administered with 3 × 10^12^ viral genomes (VG)/kg body weight. Mice treated with each vector were divided into two groups: a first group received a normal diet throughout the whole experiment and a second group alternated between chow containing 0.2% cholic acid (w/w) (CA) and normal chow for three weeks at a time. Both male and female mice inoculated with AAV-imPr-LucPEST showed a 5- to tenfold increase in luciferase expression upon transitioning to a CA diet compared with normal diet (Fig. 4a, 4c). Induction of luciferase expression was observed to recur three times coinciding with three cycles of CA diet. The luciferase signal was located in the lower thoracic/upper abdominal region of the mice, indicative of expression in liver. AAV-A1AT-LucPEST-treated mice showed no diet-dependent fluctuations in expression (Fig. 4b, 4c). In contrast to the minimal mouse promoter, AAV-ihPr-LucPEST showed very low expression levels with no diet-dependent luciferase induction in female mice (not tested in males) (Fig. 4d).

### Analysis of luciferase expression in ***Abcb4***^−/−^ mice

**Fig. 5:**
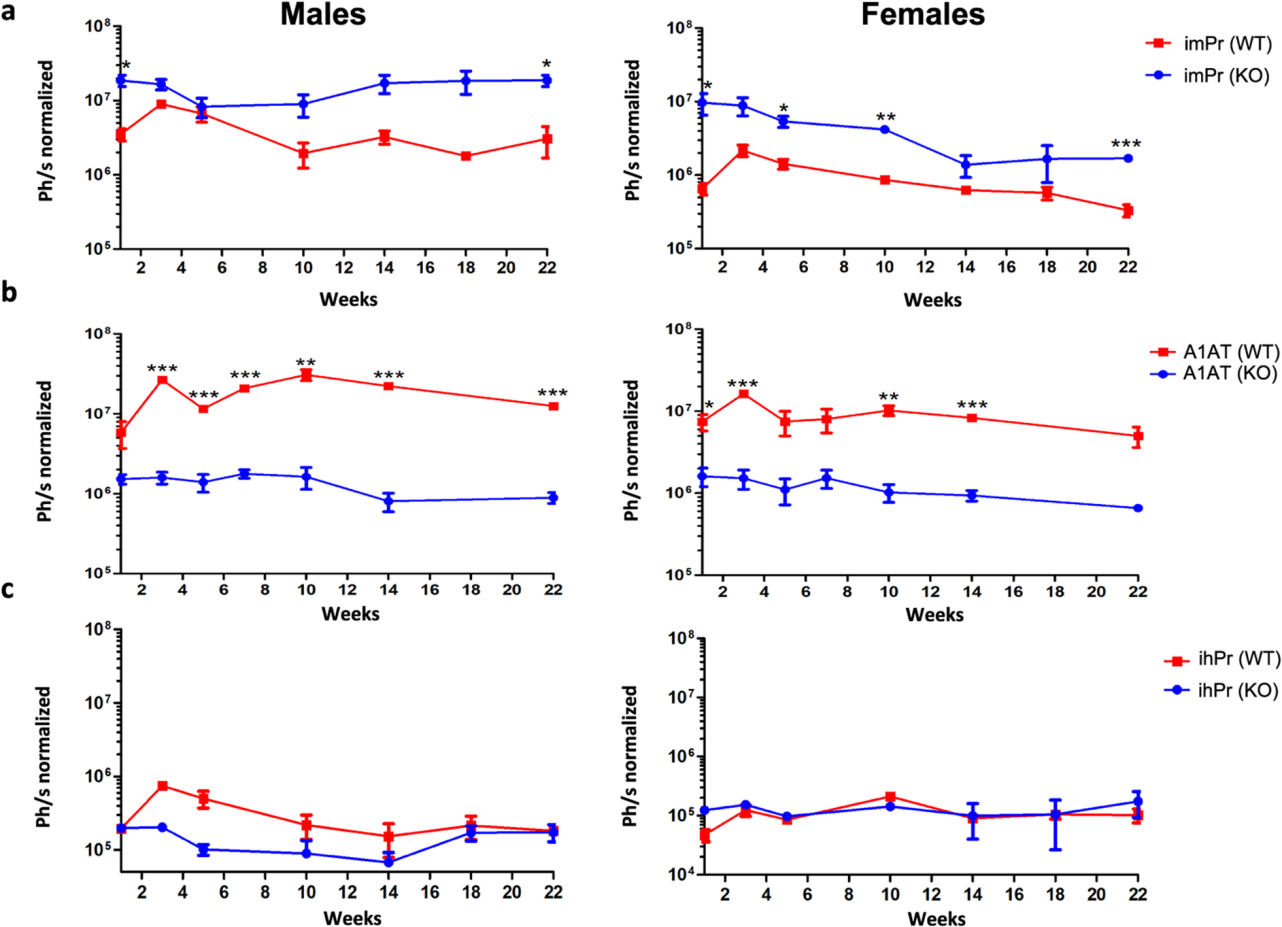
Luciferase expression in *Abcb4*^*−/−*^ mice. *Abcb4*^−/−^ mice exhibit high levels of bile acids in serum, making them a good model to test the physiological inducibility of minimal BSEP promoters. FVB *Abcb4*^*−/−*^ and WT male and female mice were administered 3 × 10^12^ VG/kg of AAV8-imPr-LucPEST (**a**), AAV8-A1AT-LucPEST (**b**) or AAV8-ihPr-LucPEST (**c**). Luciferase was measured in live mice at the indicated times (n = 3 for each gender and vector, except in knock-out (KO) males inoculated with AAV-imPr-LucPEST where n = 4). The statistical comparison between *Abcb4*^*−/−*^ and WT mice was performed by using an unpaired T test at each time point. *p < 0.05; **p < 0.01; ***p < 0.001. Data are shown as mean ± SEM of photon (Ph) units per second normalized with background signal. imPr, AAV8-imPr-LucPEST; ihPr, AAV8-ihPr-LucPEST; A1AT, AAV8-A1AT-LucPEST

*Abcb4*^−/−^ mice completely lack expression of MDR2 (the mouse ortholog of MDR3), and therefore are unable to secrete phosphatidylcholine to the bile. The lack of MDR2 results in symptoms that reproduce most of the biomarkers and pathological signs of human PFIC3, including high levels of serum transaminases and BAs compared to wild-type (WT) mice [16]. AAV vectors (AAV8-imPr-LucPEST, AAV8-ihPr-LucPEST, or AAV8-A1AT-LucPEST) were administered to four-week-old male and female WT and *Abcb4*^*−/−*^ mice at the same dose as before. AAV8-imPr-LucPEST treatment induced significantly higher levels of luciferase expression in *Abcb4*^−/−^ mice than WT mice (Fig. 5a). Moreover, in *Abcb4*^−/−^ mice AAV-imPr-LucPEST provided expression levels that were not significantly different from WT mice receiving AAV-A1AT-LucPEST vector (Fig. 5b) (p = 0.17 and p = 0.077 for males and females, respectively). However, the A1AT vector expressed significantly lower levels of luciferase in *Abcb4*^*−/−*^ mice than in WT mice, which was the opposite of what was observed with AAV-imPr-LucPEST. We observed very similar results when we quantified liver LucPEST mRNA levels by RT-qPCR (Additional file 1: Fig. S5a). However, *Abcb4*^*−/−*^ mice showed a significantly lower amount of AAV genomes than WT mice (Additional file 1: Fig. S5b). The fact that despite this detrimental effect in transduction AAV-imPr-LucPEST showed higher expression in *Abcb4*^*−/−*^ mice reinforces the idea that the high bile acid levels present in these mice can activate imPr-regulated transcription. As expected, *Abcb4*^*−/−*^ mice showed significantly higher levels of bile acids in serum when compared with WT mice (Additional file 1: Fig. S6). As observed in C57BL/6 mice, AAV-ihPr-LucPEST showed very low expression levels in vivo with no apparent differences between WT and *Abcb4*^*−/−*^ mice (Fig. 5c).

### Increasing number of IR-I motifs in imPr improves luciferase expression

**Fig. 6:**
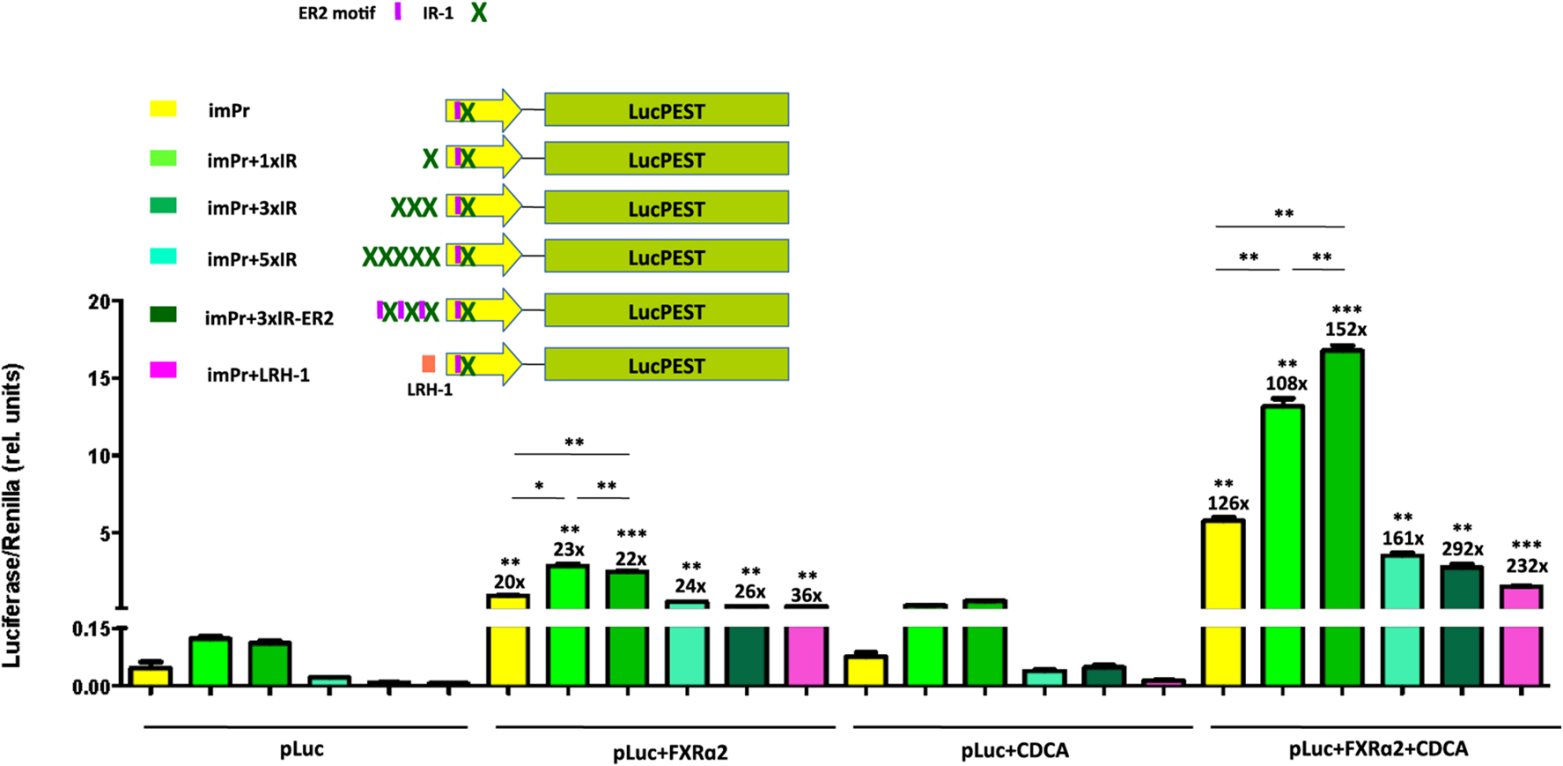
Bile acid induction of luciferase expression of optimized imPr versions. Huh-7 cells were transfected with AAV plasmids expressing LucPEST downstream of the indicated imPr promoter versions with or without co-transfection with the human FXRα2 and with or without incubation with CDCA. All samples were tested in triplicates and data are presented as mean + SEM of relative (rel.) units (Luciferase units sec^−1^/Renilla units sec^−1^). The fold induction of each condition relative to cells transfected only with the various luciferase plasmids (pLuc) is indicated on top of each column, as well as the statistical analysis comparing these two conditions. Other comparisons are shown by horizontal bars. *p < 0.05; **p < 0.01; ***p < 0.001. imPr, AAV-imPr-LucPEST; IR-1, inverted repeat (IR)-1 element; ER2, everted repeat separated by two nucleotides; LRH-1, liver receptor homolog 1 element

In an attempt to improve the inducibility and expression of the imPr promoter, we modified it by adding one (imPr + 1xIR), three (imPr + 3xIR), or five (imPr + 5xIR) additional repeats of the murine IR-1 element to its 5´end (Fig. 6). Moreover, we also designed a new imPr promoter containing three repeats of the IR-1 element bound to the ER2 motif (imPr+3xIR-ER2), a short sequence located immediately upstream of IR-1 which seems to be required for maximal transactivation of BSEP by FXRα2 [10]. Finally, we also constructed a slightly longer version of imPr including an upstream sequence for the liver receptor homolog-1 (LRH-1) element (imPr+LRH-1), a short sequence that acts as a modulator regulated by FXR [12].

We tested luciferase expression of plasmids containing the new promoters in vitro. Plasmids containing imPr+1xIR and imPr+3xIR promoters showed a significantly higher level of luciferase expression than pAAV-imPr-LucPEST in cells co-transfected with pCMV-hFXRɑ2 and incubated with CDCA (Fig. 6), indicating that the addition of one or three extra IR-1 sequences can enhance bile acid-induced expression with the degree of improvement dependent on the number of IR-1 repeats. Despite their higher expression, the inducibility of these new promoters was comparable to imPr, since they showed higher uninduced baseline expression levels (Fig. 6). Plasmids containing imPr+5xIR, imPr+3xIR-ER2, and imPr+LRH-1 promoters did not show improved expression compared to imPr.

### An imPr with three extra IR-1 repeats shows higher inducible expression in WT mice receiving a BA-enriched diet

**Fig. 7:**
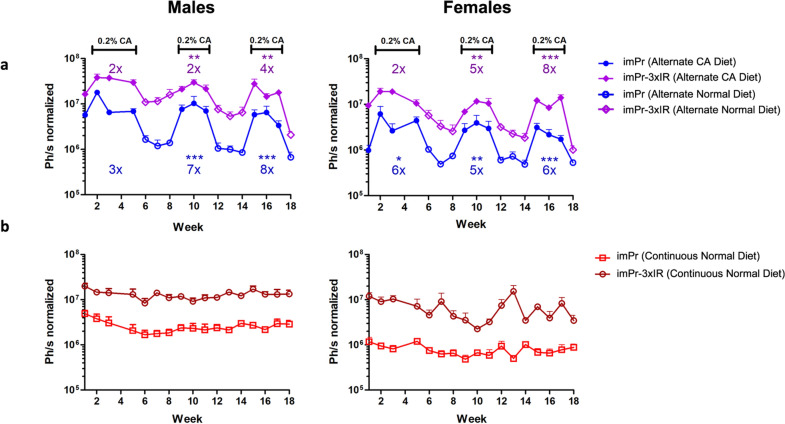
BA induction of an optimized minimal BSEP promoter in wild type mice. C57BL/6 male and female mice were administered 3 × 10^12^ VG/kg of AAV8-imPr-3xIR-LucPEST or AAV8-imPr-LucPEST and received either a diet supplemented with 0.2% CA (closed symbols) alternating with normal diet (open symbols) (**a**) or a continuous normal diet (**b**). Luciferase was measured in live mice at the indicated times (n = 4). Data are shown as mean + SEM of photon (Ph) units per second normalized to background signal. The statistical comparisons and fold induction were calculated as described in Fig. 4a (indicated in the upper part of the graph for imPr-3xIR and in the lower part for imPr). imPr, AAV8-imPr-LucPEST; imPr-3xIR, AAV8-imPr-3xIR-LucPEST

Next, four-week-old C57BL/6 WT male and female mice were administered intravenously with 3 × 10^12^ VG/kg AAV8-imPr-LucPEST or AAV8-imPr-3xIR-LucPEST and submitted to cycles of CA diet supplementation as described before. Mice treated with AAV-imPr-3xIR-LucPEST showed a two–eightfold increase in luciferase expression during each CA diet cycle compared to uninduced baseline levels (Fig. 7a). The luciferase signal was in a region indicative of expression in liver. No relevant differences between males and females were observed in the degree of induction, but luciferase expression was again slightly higher in males. Upon induction, the expression levels obtained with AAV8-imPr-3xIR-LucPEST were higher than with AAV-imPr-LucPEST (2–ninefold increase) confirming the in vitro results. Baseline levels of mice on normal diet that received AAV-imPr-3xIR-LucPEST were also higher than for AAV-imPr-LucPEST (Fig. 7b).

## Discussion

AAV-based gene therapy for monogenic diseases has become a successful therapeutic innovation with several products already approved for clinical use and with many others showing very promising results in clinical trials [19, 20]. In most of these vectors, transgene expression is driven by constitutive promoters of small size, since the AAV cloning capacity is limited [14]. An interesting possibility for gene therapy of some diseases, such as metabolic disorders, is the use of vectors that utilize endogenous regulation of transgene expression. With this type of vectors, the level of expression would ideally increase or decrease with the same kinetics as the endogenous gene that is being restored, limiting potential side effects associated with protein overexpression. In the present study we have addressed the possibility of achieving such endogenous regulation by using minimal versions of the BSEP promoter, which is sensitive to the level of BAs. This kind of inducible system could highly benefit gene therapy for genetic cholestatic diseases, like PFIC2 and PFIC3, in which the affected transgenes have large promoters, which prohibit their incorporation into delivery vectors with limited capacity such as AAV, that are physiologically regulated by BAs.

Although the full-length BSEP-Pr is too large for its incorporation into AAV-based gene therapy vectors, we demonstrated that minimal versions of this promoter of only 150 nt can efficiently transactivate reporter gene expression upon induction with the FXR agonist chenodeoxycholic acid (CDCA). Interestingly, the minimal murine promoter (imPr) showed a significantly higher expression compared to the minimal human promoter (ihPr) or to the full-length BSEP promoters of either species in human and murine hepatic cells (Fig. 2, 3 and Additional file 1: Figs. S3, S4). In these experiments, optimal induction was obtained when cells were co-transfected with a second plasmid expressing human FXR isoforms, confirming that hepatic cell lines do not express sufficient amounts of this transcription factor [4, 7, 12, 21]. Transfection of FXR without addition of CDCA led to a low degree of luciferase induction, which could be due to the fact that hepatic cells can express low levels of BAs. An alternative explanation is that overexpressed FXR could bind to the minimal promoter, inducing transgene expression in the absence of BAs [22]. Although both FXRɑ1 and FXRɑ2 isoforms were able to transactivate the reporter gene under control of minimal promoters in murine cells, FXRɑ1 failed to activate ihPr in human cells (Additional file 1: Fig. S4). It has been described that human BSEP is predominantly regulated by FXRɑ2 over FXRɑ1, while in mice BSEP can be similarly transactivated by the two isoforms [10]. Based on evidence that FXR recognizes the IR-1 response element present in BSEP promoters, we were able to significantly improve the performance of imPr by adding several repeats of this short DNA sequence at its 5´end (Fig. 6). This approach has also been used to increase the inducibility of other minimal promoters like the one controlling interferon (IFN) stimulated gene MxA. By adding three interferon-sensitive response element (IRF-ISRE) repeats at the 5´end, Nistal-Villan et al. showed that IFN-mediated induction of a reporter gene could be potentiated [23]. As in the case with imPr, the addition of more than three repeats was not beneficial and led to lower expression levels. It is possible that by adding too many copies of the response element at the 5´end, transcription factors are unable to bind with the same affinity, kinetics or localization as they do to the endogenous promoter, leading to reduced transactivation.

To evaluate whether AAV vectors with a minimal BSEP-Pr could be regulated in vivo by BAs we set up a mouse study based on cyclic administration of a CA-enriched diet. We used AAV8 vectors due to their high tropism for the liver [24], and expression was localized in that organ (Fig. 4c). The vector in which luciferase expression was driven by imPr showed the capability to be reinduced repeatedly upon supplementation with CA, with sharp drops in expression after removing CA from the diet (Figs. 4, 7). As observed in vitro, an optimized imPr version with three extra IR-1 repeats (imPr + 3xIR) led to higher luciferase expression in vivo compared to imPr. Interestingly, this optimized promoter also led to a higher baseline expression both in vitro and in vivo, which could be beneficial in situations in which a higher basal level of transgene expression is required, even in the absence of the inducer. An unexpected result observed in vivo was the almost complete absence of activity of ihPr, which resulted in very low luciferase expression regardless of BA levels (Fig. 4d). Although we do not have a clear explanation for this observation it is possible that murine FXR isoforms are not able to efficiently transactivate this human minimal promoter. However, imPr seems to be efficiently transactivated by human FXR isoforms (Fig. 3, 4 and Additional file 1: Figs. S3, S4).

In order to test the functionality of this type of promoter in a pathological situation with altered BA homeostasis, we tested our AAV reporter vectors in *Abcb4*^*−/−*^ mice, which have high circulatory levels of BAs (Additional file 1: Fig. S6) and are considered a good surrogate model for PFIC3 [25]. Remarkably, imPr led to significantly higher luciferase levels in KO mice compared to WT mice, indicating that the higher BA levels present in these mice may have resulted in the transactivation of this promoter. Most interestingly, this higher expression was achieved despite lower liver transduction in KO mice (Additional file 1: Fig. S5b), which was mostly due to liver fibrosis, as consequence of the disease [16, 25–27]. An opposite situation was observed with a control vector expressing luciferase from a constitutive promoter that showed a significantly lower expression in KO mice.

## Conclusions

Here we present novel gene therapy transgene construct elements using vectors with BA-inducible promoters that function with physiological transgene regulation providing potential as a therapeutic approach to cholestatic diseases with BA alterations, such as PFIC2 and PFIC3.

## Methods

### Cell lines

Human hepatoma cells HuH-7 (JCRB0403-A, JCRB Cell Bank, Japan) and HepG2 (ATCC® HB-8065), murine hepatoma cells Hepa 1–6 (C0015005, AddexBio, San Diego, CA), and HEK-293T (T0011002, AddexBio) cells were grown in DMEM (Gibco BRL) supplemented with 10% fetal bovine serum (FBS), 2 mM glutamine, and 100 μg/mL streptomycin and 100 U/mL penicillin.

### Construction of AAV plasmids

Synthetic DNA sequences of minimal BSEP promoters from mouse (imPr) and human (ihPr) origin (Additional file 1: Fig. S1) were based on human and mouse full-length BSEP promoters, respectively (NCBI Reference Sequences: AF190696.1 and AF190697.1, respectively) and obtained from GenScript (Nanjing, China) cloned into pUC57 flanked by Mlu I and Nhe I sites (pUC57-imPr & pUC57-ihPr). To generate AAV plasmids expressing luciferase, we used plasmid pAAV-A1AT-LucPEST (kindly provided by Dr. Tomas Aragon, Cima Universidad de Navarra, Spain), which contains AAV2 ITRs flanking the destabilized firefly luciferase sequence (LucPEST, GenBank accession number AY603759) downstream of the alpha-1 antitrypsin (A1AT) promoter. The DNA fragment containing each minimal promoter was extracted from pUC57-imPr and pUC57-ihPr by digestion with Mlu I and Nhe I and subcloned into pAAV-A1AT-LucPEST substituting the A1AT promoter by ihPr and imPr, generating in this way plasmids pAAV-ihPr-LucPEST and pAAV-imPr-LucPEST, respectively. The sequences of imPr in which one, three or five extra tandem repeats of the murine IR-1 element (TTAGGCCATTGACCTA), or three extra tandem repeats of the IR-1 element preceded by the ER2 motif (TGGACT) at the 5´end were ordered to Genscript and subcloned into pAAV-imPr-LucPEST plasmid replacing the imPr promoter using Kpn I and Sac I restriction sites, generating plasmids pAAV-imPr+1xIR-LucPEST, pAAV-imPr+3xIR-LucPEST, pAAV-imPr+5xIR-LucPEST, and pAAV-imPr+3xIR-ER2-LucPEST, respectively. An extended version of imPr including the liver receptor homolog-1 (LRH-1) element (TTTCTAAAGCT) at its 5´ end was also ordered to GenScript and subcloned into pAAV-imPr-LucPEST as described above, generating plasmid pAAV-imPr+LRH-1-LucPEST.

To generate pAAV-mBSEPpr-LucPEST, a 2488 nt synthetic sequence of full-length mouse BSEP promoter (NCBI AF190697.1) was also ordered from GenScript cloned into pUC57 flanked by Mlu I and Nhe I sites and used to substitute A1AT promoter in pAAV-A1AT-LucPEST, as described before (Additional file 1: Fig. S2a). An additional control vector containing the luciferase gene downstream of a full-length human BSEP promoter of 2563 nt (p-2563/+4-Luc) (Additional file 1: Fig. S2b) was kindly provided by Dr. James Boyer (Yale University, New Haven, CT). Finally, a plasmid expressing human FXRɑ1 (pcDNA3.1-hFXRα1) was ordered from GenScript and a plasmid expressing human FXRɑ2 (pCMV-hFXRɑ2) was obtained from cDNA Resource Center (Bloomsburg, PA).

### Analysis of luciferase expression in vitro

Twelve-well plates were seeded with human (HepG2 or Huh-7) or mouse (Hepa 1-6) hepatic cells on day 0 with 5 × 10^5^ cells/well and co-transfected the next day with 0.5 µg/well of the plasmid expressing luciferase to be tested and 0.1 µg/well of the plasmid expressing the selected FXR isoform using lipofectamine 2000 (Thermo Fisher, Waltham, Massachusetts) at a DNA/lipofectamine ratio of 1:3. To control the transfection efficiency, cells were co-transfected with 0.1 µg/well of a plasmid expressing Renilla-luciferase from a constitutive promoter (pRL-CMV, AF025843). At 24 h post-transfection, cells were incubated with 30 µM (human cells) and 100 µM (mouse cells) chenodeoxycholic acid (CDCA, Sigma-Aldrich, St. Louis, MO), or 0.3% and 1.0% DMSO (Sigma-Aldrich) as controls, for 30 h. The amount of CDCA used to induce each specific cell line was previously optimized (data not shown). The expression of the Renilla/firefly luciferase system was determined from cell lysates using a non-commercial dual luciferase enzyme assay as described Dyer et al. [28] and the results were measured with an Orion L Microplate Luminometer (Berthold Technologies, Germany).

### Production of AAV vectors

To produce AAV8 viral particles (VPs), 150 cm^2^ flasks containing confluent HEK-293T cells were cotransfected, using 25 kDa linear polyethyleneimine (Polysciences, Warrington, PA), with the AAV plasmid of interest and pDP8.ape (Plasmid Factory, Germany), which contains adenoviral helper genes plus AAV2 rep and AAV8 cap genes. After 72 h, the supernatant was collected, treated with 8% v/v polyethylene glycol and incubated for 48–72 h at 4 °C. The supernatant was centrifuged at 1378*g* for 15 min and the pellet was resuspended in lysis buffer (50 mM Tris–Cl, 150 mM NaCl, 2 mM MgCl2, 0.1% Triton X-100) and stored at − 80 °C. Cells were also collected, treated with lysis buffer, and frozen at − 80 °C. After three cycles of freezing and thawing, the VPs obtained from the supernatants and cell lysates were purified by ultracentrifugation at 350,000*g* for 2.5 h in a 15–57% iodioxanol gradient. Finally, the purified virus was concentrated using Amicon Ultracel 100 K ultra centrifugal filters (Millipore). The AAV titers (VGs/mL) were determined by quantitative PCR (qPCR). VGs treated with DNase were extracted from VPs using the High Purity Viral Nucleic Acid Kit (Roche, Switzerland) and specific primers for LucPEST (Fw: 5′-TCTGAGGAGCCTTCAGGATT-3′ and Rv: 5′-TTTTGGCGAAGAAGGAGAAT-3′) and ITRs (Fw: 5′-GGAACCCCTAGTGATGGAGTT-3′ and Rv: 5′-CGGCCTCAGTGAGCGA-3′) were used for qPCR.

### Animal studies

FVB Mdr2^−/−^ mice (FVB.129P2-Abcb4tm1Bor; JAX stock #002539) (*Abcb4*^*−/−*^) [16] and FVB Mdr2^+/+^ (*Abcb4*^+*/*+^) mice (The Jackson Laboratory, Bar Harbor, ME) were raised in our own facilities (Cima Universidad de Navarra, Spain). C57BL/6 wild-type mice were purchased from Envigo (Spain). Mice were housed on a 12 h light–dark cycle and received water and food ad libitum using a standard food or a special diet according to the study. Treatment with AAV vectors was performed in male and female mice at four weeks of age by retroorbital injection. For serum analysis, mice were bled intravenously at weeks 1, 3, 5, 10, 14, 18, and 22 post-inoculation. Serum was separated from whole blood by centrifuging at 2300×*g* for 15 min in a microfuge. Total bile acids were quantified using a HITACHI C311 analyzer (Roche). In some experiments C57BL/6 mice were given a special diet containing 0.2% (w/w) sodium cholate (Sigma-Aldrich). This diet was administered in three-week periods alternating with three-week periods of normal diet, for a total of three cycles. All animal experiments and procedures were carried out in accordance with the ethical standards for animal experimentation and the studies were reviewed and approved by the Institutional Ethical Committee of the University of Navarra (protocol numbers: 082c-17 for reproduction, 086-17 and 024-18 for animal studies).

### Vector genome and transgene expression quantification

Vector genome copies and LucPEST mRNA present in liver extracts were determined by qPCR using iQ™ SYBR® Green (BioRad, Hercules, CA) in a CFX96 Real-Time Detection System (BioRad) with primers specific for LucPEST (Fw: 5′-TCTGAGGAGCCTTCAGGATT-3′ and Rv: 5′-TTTTGGCGAAGAAGGAGAAT-3′). Mouse GAPDH was used as a normalizing gene (Fw: 5′-GGATGCAGGGATGATGTTC-3′ and Rv: 5′-TGCACCACCAACTGCTTA-3′).

### Bioluminescence imaging of mice

For in vivo quantification of luciferase activity, mice were anesthetized via intraperitoneal injection with a mixture of ketamine, physiological saline and xylazine (in a ratio 3:2:3) and received an intraperitoneal injection of 100 μL d-luciferin potassium-salt substrate (Promega, Madison, Winconsin) dissolved in PBS at a final concentration of 30 µg/mL. Light emission was measured using a CCD luminometric camera (Biospace Lab, France). Photon counts were acquired 5 min after substrate administration. Light surface images were obtained immediately after each photon counting session to provide an anatomical view of the animals. Image processing and signal intensity quantifications were performed using M3 Vision software (Biospace Lab). Images are displayed as a pseudo-color photon count image, superimposed on a gray scale anatomic white-light image, allowing assessment of both bioluminescence intensity and its anatomical source. The number of photons emitted per second was calculated as a measure of luciferase activity utilizing a constant region of interest.

### Statistical analysis

Data are presented as mean values ± standard error of the mean (SEM) and were statistically analysed using unpaired or paired t test with GraphPad Prism 5.01 software (GraphPad Software Inc., San Diego, CA) considering P < 0.05 significant.

## Supplementary Information

**Additional file 1: Figure S1.** Nucleotide sequences of minimal BSEP derived promoters. **Figure S2.** Diagrams of vectors expressing luciferase downstream of full-length BSEP promoters. **Figure S3.** Bile acid induction of LucPEST expression in Huh-7 cells co-transfected with human FXRα2 isoform. **Figure S4.** Bile acid induction of luciferase expression in Huh7 cells co-transfected with human FXRα1 isoform. **Figure S5.** AAV transduction and transgene expression for *Abcb4*^*−/−*^ and WT mice treated with AAV8-LucPEST. **Figure S6**. Bile salt levels in *Abcb4-/-* and WT mice.

## Data Availability

The datasets used and/or analyzed during the current study are available from the corresponding author on reasonable request.

## Abbreviations

A1AT: Alpha-1 antitrypsin
AAV: Adeno-associated virus
BA: Bile acid
BSEP: Bile salt excretory pump
BSEP-Pr: BSEP promoter
CA: Cholic acid
CDCA: Chenodeoxycholic acid
CYP7A1: Cholesterol 7α-hydroxylase
ER2: Everted repeat separated by two nucleotides
FBS: Fetal bovine serum
FIC1: Familial intrahepatic cholestasis 1
FXR: Farnesoid X receptor
FXRE: Farnesoid X receptor response element
IFN: Interferon
ihPr: Minimal human inducible BSEP promoter
imPr: Minimal mouse inducible BSEP promoter
IR-1: Inverted repeats separated by one nucleotide
IRF-ISRE: Interferon-sensitive response element
ITR: Inverted terminal repeat
LRH-1: Liver receptor homolog-1
LRHRE: Liver receptor homolog-1 responsive element
LucPEST: Destabilized firefly luciferase
MDR3: Multidrug resistance protein 3
PFIC2: Progressive familial intrahepatic cholestasis 2
qPCR: Quantitative Polymerase chain reaction
RXR: Retinoic acid receptor
SEM: Standard error of the mean
SHP: Small heterodimer partner
UTR: Untranslated region
VPs: Viral particles
VGs: Viral genomes
WT: Wild-type
