# Supplementary material for: A minimal bile salt excretory pump promoter allows bile acid-driven physiological regulation of transgene expression from a gene therapy vector
Source: Cell Biosci. 2022 May 31;12:79. doi: 10.1186/s13578-022-00803-9 (PMC9158313; doi:10.1186/s13578-022-00803-9)
Supplement: Supplementary file 1 — Additional file 1: Figure S1. Nucleotide sequences of minimal BSEP derived promoters. Figure S2. Diagrams of vectors expressing luciferase downstream of full-length BSEP promoters. Figure S3. Bile acid induction of LucPEST expression in Huh-7 cells co-transfected with human FXRα2 isoform. Figure S4. Bile acid induction of luciferase expression in Huh7 cells co-transfected with human FXRα1 isoform. Figure S5. AAV transduction and transgene expression for Abcb4−/− and WT mice treated with AAV8-LucPEST. Figure S6. Bile salt levels in Abcb4-/- and WT mice. [file 13578_2022_803_MOESM1_ESM.pdf]

a

ihPr from nt -145 to + 86

TTCCCAAGCACACTCTGTGTTTGGGGTTATTGCTCTGAGTATGTTTCTCGTATGTCACTGAACTGTGCTTG

ER2 motif FXRE (IR-1) HNF-5 TATA-like

GGCTGCCCTTA**GGGACATTGATCCT**TAGGCAAATAGATAATGTTCTTGAAAAAGTTTGAATTCTGTTCAGT

+1 5'-UTR

GCTttagaatgatgaaaaccgaggttggaaaaggttgtgaaacctttttaactctccacagtggagtccatt

atttcctctggcttcctc

b

imPr from nt -145 to + 77

ER2 motif FXRE

GGTTCCTGCTTTGAGTATGTTCGACCTTTCCTCTCATGTCACTGAACTGTGCTAGATCTGGACTTT**AGGCC**

(IR-1) HNF-5

ATTGACCTATAAGCAAATAGATAGTGTTCTTAAAAAAGCCTGATTTCTGTTCAATGCTTTATTACCATGAA

+1 5'-UTR

AACtgaacttggaaaggggtgtacaaccctgactttccacagtggcgtctctcgcttctcctggctccctc

aaattcaca

Figure S1. Nucleotide sequences of minimal BSEP derived promoters. The sequences of minimal BSEP promoters from human (a) and mouse (b) origin used to generate pAAV-ihPr-LucPEST and pAAV-imPr-LucPEST, respectively are shown. Numbering of nucleotides is relative to the transcription start site (nt +1, arrow). Transcriptional regulatory sequences are underlined, and their cognate acting factors are indicated in boldface type. FXRE, Farnesoid- X-receptor element, IR-1, inverted repeat-1 element; ER2, everted repeat separated by two nucleotides motif.

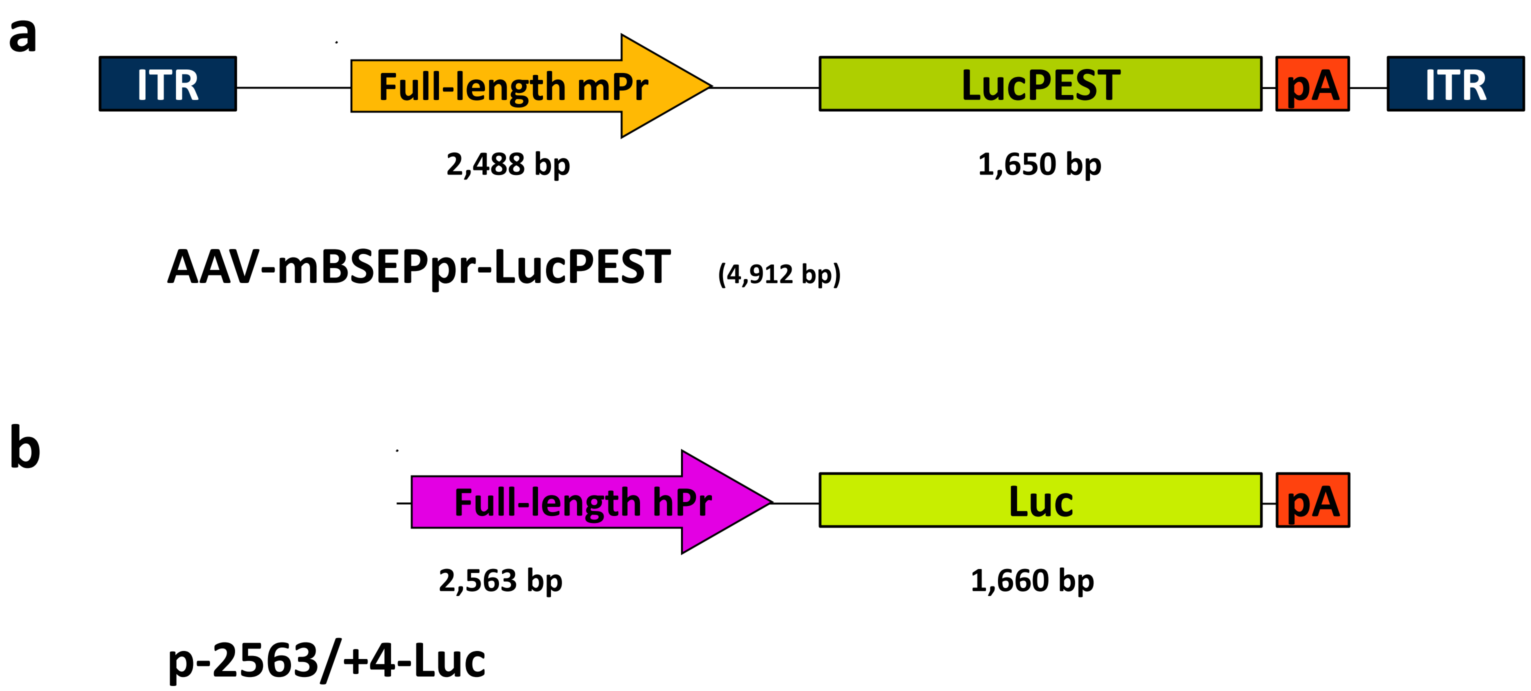

Figure S2. Diagrams of vectors expressing luciferase downstream of full-length BSEP promoters. Schematic representation of endogenous mouse full-length BSEP promoters (mPr) (a) and human full-length hPr (b). ITR, inverted terminal repeats; LucPEST, destabilized firefly luciferase; Luc, firefly luciferase; pA, polyadenylation signal.

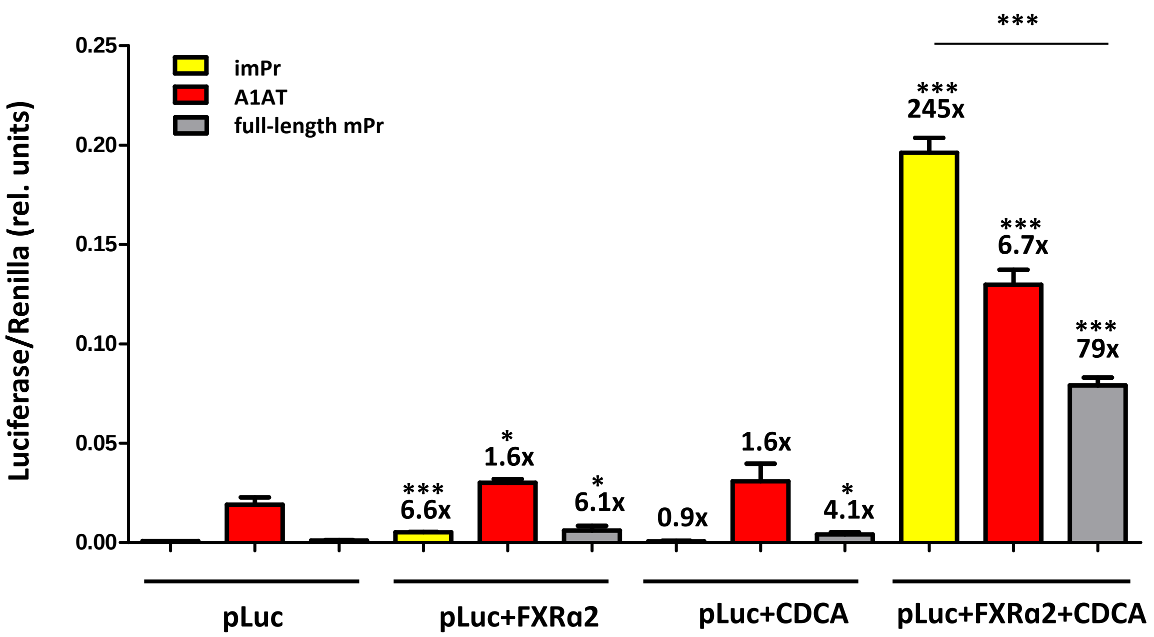

Figure S3. Bile acid induction of LucPEST expression in Huh-7 cells co-transfected with human FXRα2 isoform. Huh-7 cells were transfected with plasmids expressing luciferase downstream of the indicated promoters with or without the human FXRα2 isoform and with or without incubation with CDCA. All samples were tested in triplicates and data are presented as mean + SEM of relative (rel.) units (Luciferase units sec^{-1}/Renilla units sec^{-1}). The fold induction of each condition relative to cells transfected only with the various luciferase plasmids (pLuc) is indicated on top of each column, as well as the statistical analysis comparing these two conditions. Other comparisons are shown by horizontal bars. *, p<0.05; p<0.01; ***, p<0.001; ns, not significant. imPr, pAAV-imPr-LucPEST; full-length mPr, pAAV-mBSEPpr-LucPEST; A1AT, pAAV-A1AT-LucPEST.

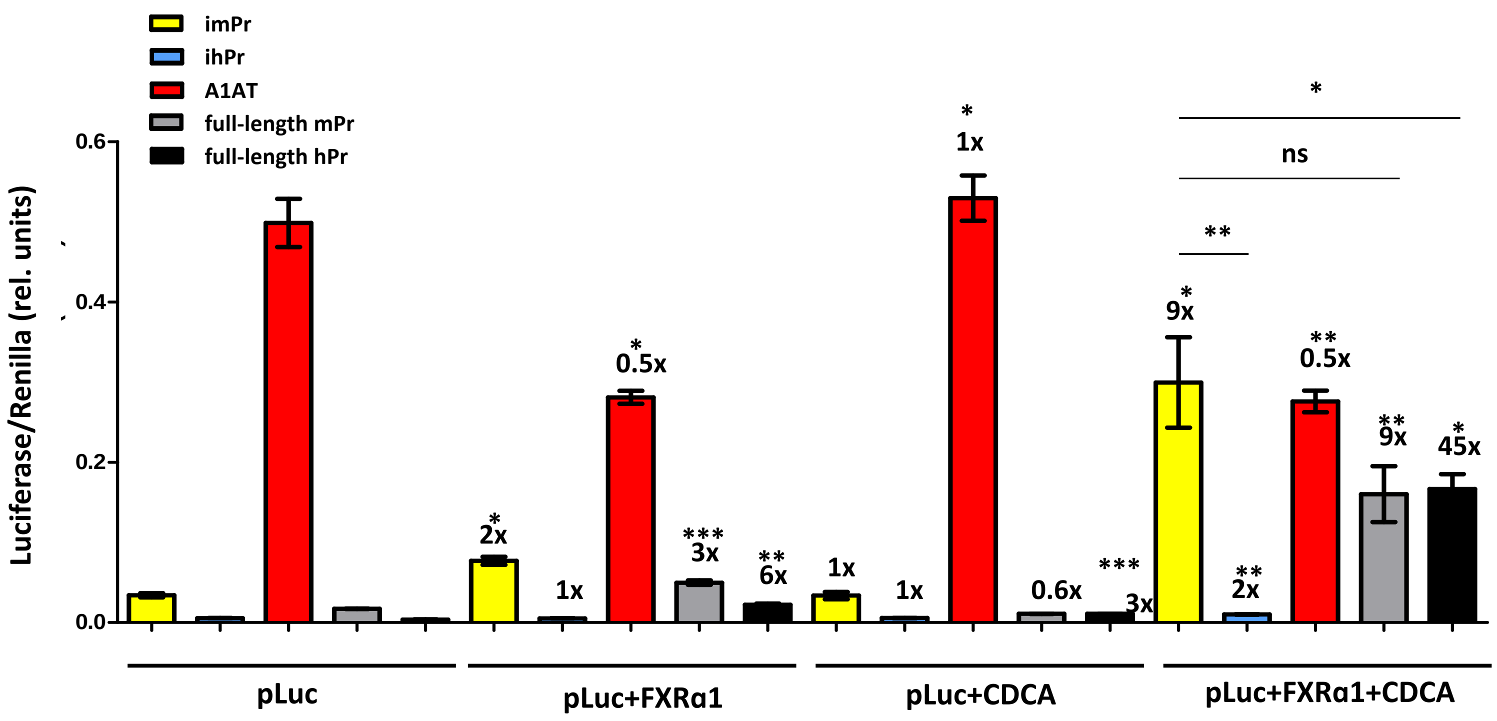

Figure S4. Bile acid induction of luciferase expression in Huh7 cells co-transfected with human FXRα1 isoform. Huh-7 cells were transfected with plasmids expressing luciferase downstream the indicated promoters with or without the human FXRα1 isoform and with or without incubation with CDCA. All samples were tested in triplicates and data are presented as mean ± SEM of relative (rel.) units (Luciferase units sec^{-1}/Renilla units sec^{-1}). The fold induction of each condition relative to cells transfected only with the various luciferase plasmids (pLuc) is indicated on top of each column, as well as the statistical analysis comparing these two conditions. Other comparisons are shown by horizontal bars. *, $p<0.05$; **, $p<0.01$; ***, $p<0.001$; ns, not significant. imPr, pAAV-imPr-LucPEST; ihPr, pAAV-ihPr-LucPEST; full-length mPr, pAAV-mBSEPpr-LucPEST; full-length hPr, p-2563/+4-Luc; A1AT, pAAV-A1AT-LucPEST.

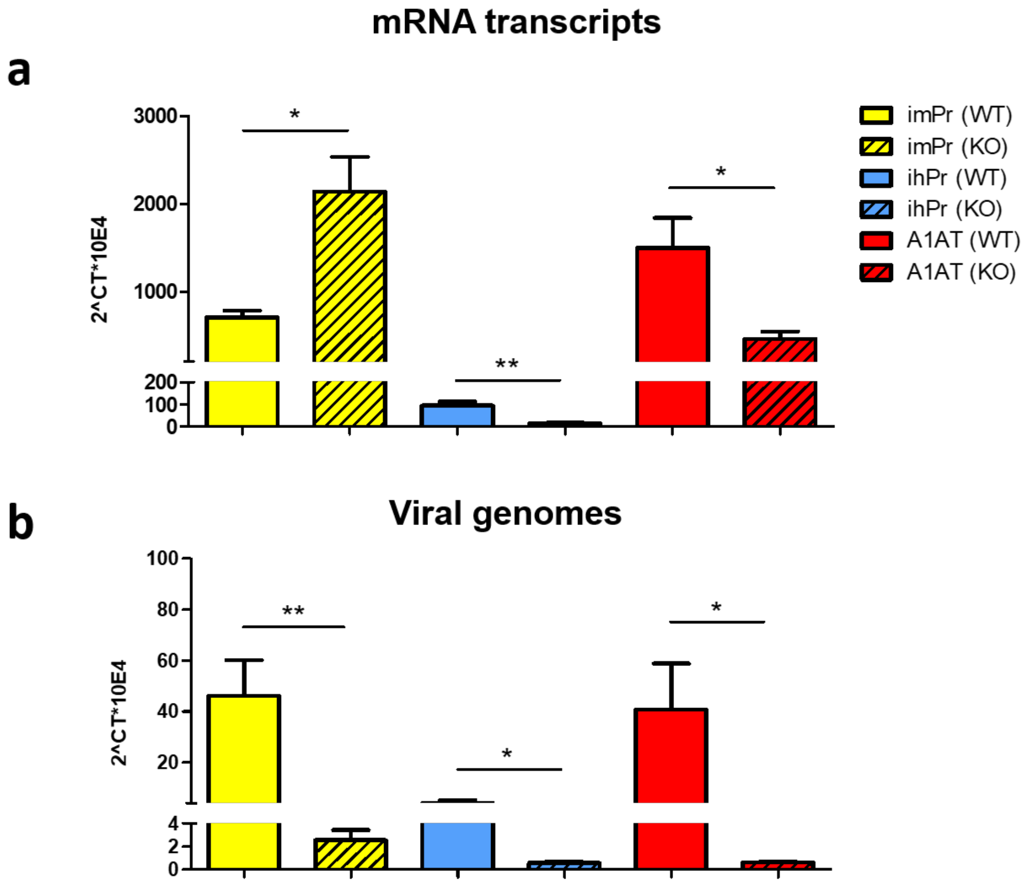

Figure S5. AAV transduction and transgene expression for *Abcb4*$^{-/-}$ and WT mice treated with AAV8-LucPEST. The levels of LucPEST mRNA transcripts (a) and AAV genomes (b) were quantified from liver tissue harvested from *Abcb4*$^{-/-}$ and WT mice treated with AAV8-imPr-LucPEST, AAV8-ihPr-LucPEST or AAV8-A1AT-LucPEST at 3×10^{12} VG/kg. AAV genomes and LucPEST transcripts were quantified via qPCR and RT-qPCR, respectively. Animals were sacrificed at 22 weeks after treatment. The graphs show the mean + SEM values of all mice, including males and females (since no significant differences were observed between genders). *, $p<0.05$; **, $p<0.01$. imPr, AAV8-imPr-LucPEST; ihPr, AAV8-ihPr-LucPEST; A1AT, AAV8-A1AT-LucPEST.

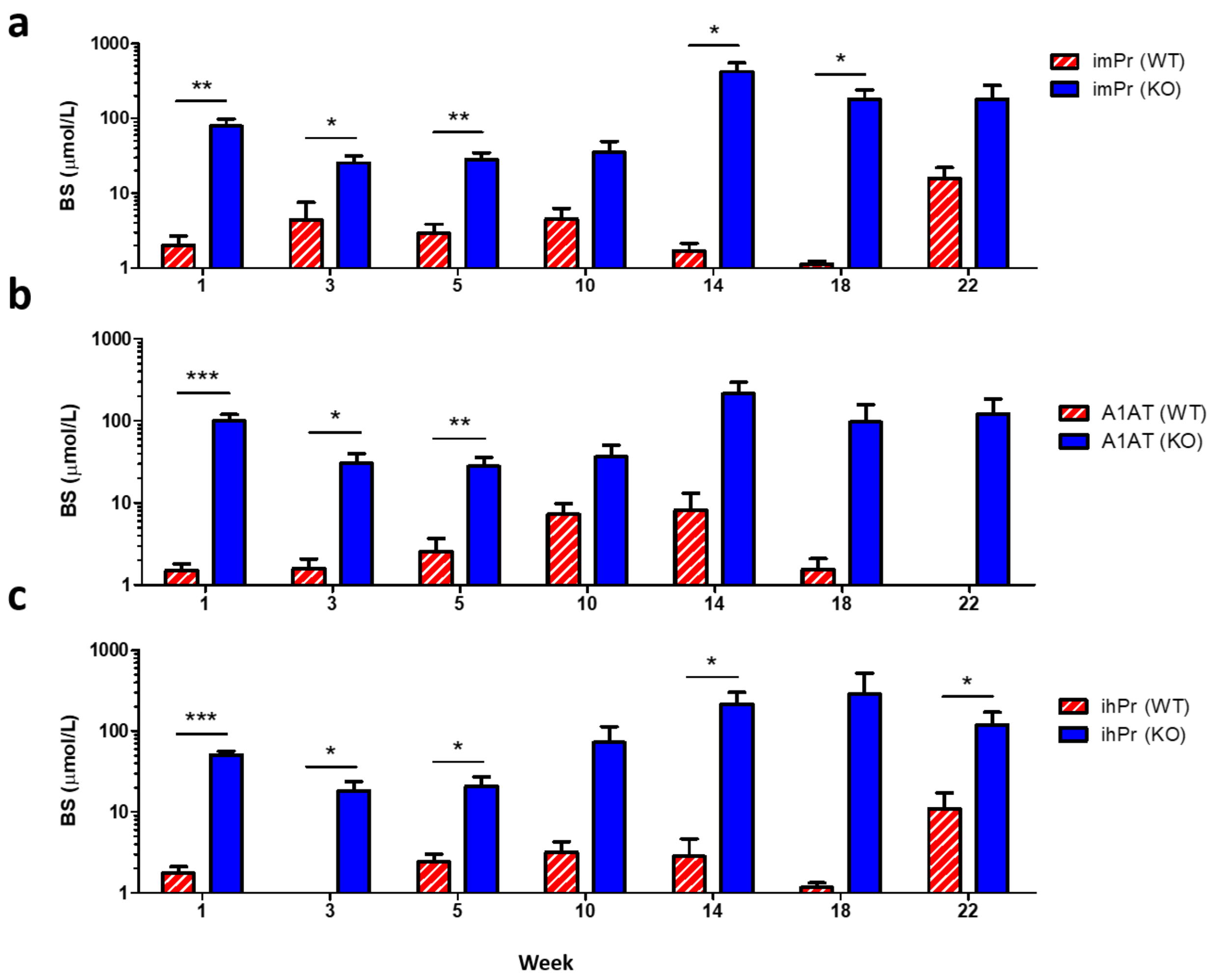

Figure S6. Bile salt levels in *Abcb4*$^{-/-}$ and WT mice. Mice were bled at the indicated times post-injection and the content of total bile salts (BS) in serum was measured. The graphs show the mean + SEM values of all mice (including males and females since no significant differences were observed between genders), treated with $3x10^{12}$ VG/kg of AAV8-imPr-LucPEST (a), AAV8-A1AT-LucPEST (b), or AAV8-ihPr-LucPEST (c). *, p<0.05; **, p<0.01; ***, p<0.001. imPr, AAV8-imPr-LucPEST; ihPr, AA8V-ihPr-LucPEST; A1AT, AAV8-A1AT-LucPEST.
